# Salivary and fecal microbiota: potential new biomarkers for early screening of colorectal polyps

**DOI:** 10.3389/fmicb.2023.1182346

**Published:** 2023-08-16

**Authors:** Limin Zhang, Ziying Feng, Yinghua Li, Cuiting Lv, Chunchun Li, Yue Hu, Mingsheng Fu, Liang Song

**Affiliations:** ^1^Department of Stomatology, Shanghai Fifth People’s Hospital, Fudan University, Shanghai, China; ^2^Central Laboratory, Shanghai Fifth People’s Hospital, Fudan University, Shanghai, China; ^3^Department of Gastroenterology, Shanghai Fifth People’s Hospital, Fudan University, Shanghai, China

**Keywords:** colorectal polyp, salivary microbiota, fecal microbiota, full-length 16S rRNA sequencing, biomarker

## Abstract

**Objective:**

Gut microbiota plays an important role in colorectal cancer (CRC) pathogenesis through microbes and their metabolites, while oral pathogens are the major components of CRC-associated microbes. Multiple studies have identified gut and fecal microbiome-derived biomarkers for precursors lesions of CRC detection. However, few studies have used salivary samples to predict colorectal polyps. Therefore, in order to find new noninvasive colorectal polyp biomarkers, we searched into the differences in fecal and salivary microbiota between patients with colorectal polyps and healthy controls.

**Methods:**

In this case–control study, we collected salivary and fecal samples from 33 patients with colorectal polyps (CP) and 22 healthy controls (HC) between May 2021 and November 2022. All samples were sequenced using full-length 16S rRNA sequencing and compared with the Nucleotide Sequence Database. The salivary and fecal microbiota signature of colorectal polyps was established by alpha and beta diversity, Linear discriminant analysis Effect Size (LEfSe) and random forest model analysis. In addition, the possibility of microbiota in identifying colorectal polyps was assessed by Receiver Operating Characteristic Curve (ROC).

**Results:**

In comparison to the HC group, the CP group’s microbial diversity increased in saliva and decreased in feces (*p* < 0.05), but there was no significantly difference in microbiota richness (*p* > 0.05). The principal coordinate analysis revealed significant differences in β-diversity of salivary and fecal microbiota between the CP and HC groups. Moreover, LEfSe analysis at the species level identified *Porphyromonas gingivalis, Fusobacterium nucleatum, Leptotrichia wadei, Prevotella intermedia,* and *Megasphaera micronuciformis* as the major contributors to the salivary microbiota, and *Ruminococcus gnavus, Bacteroides ovatus, Parabacteroides distasonis, Citrobacter freundii,* and *Clostridium symbiosum* to the fecal microbiota of patients with polyps. Salivary and fecal bacterial biomarkers showed Area Under ROC Curve of 0.8167 and 0.8051, respectively, which determined the potential of diagnostic markers in distinguishing patients with colorectal polyps from controls, and it increased to 0.8217 when salivary and fecal biomarkers were combined.

**Conclusion:**

The composition and diversity of the salivary and fecal microbiota were significantly different in colorectal polyp patients compared to healthy controls, with an increased abundance of harmful bacteria and a decreased abundance of beneficial bacteria. A promising non-invasive tool for the detection of colorectal polyps can be provided by potential biomarkers based on the microbiota of the saliva and feces.

## 1. Introduction

Colorectal polyp is a common condition of the digestive tract, generally characterized by the absence of any specific clinical manifestations, but it can cause frequent bowel movements, urgency to defecate, blood in stool, abdominal pain, and other clinical symptoms (Monreal-Robles et al., 2021; Lane and Dolejs, 2022). It can be divided into many pathological types, including adenomatous, inflammatory, proliferative, hamartoma, and lipoma, of which adenomatous polyps are the most common (Mareth et al., 2022). Colorectal adenoma is considered the precancerous lesion of colorectal cancer (CRC), which has formed the array of “adenoma-cancer” (La Vecchia and Sebastián, 2020; Zhu et al., 2020; Sahin et al., 2021). Ranking second in terms of mortality and third in terms of incidence among all cancers, CRC is a serious public health concern (Sung et al., 2021). Data released in 2021 in China, the CRC’s mortality and morbidity rates ranked third and fifth, respectively, among malignant tumors, and it is a serious threat to human health and safety (Sun et al., 2021). Research has shown that 85% cases of CRC had a history of colorectal adenoma; however, the process of malignant transformation of adenoma into adenocarcinoma may take approximately 8–15 years (Corley et al., 2014; Click et al., 2018). There are no overt symptoms during the early phase of CRC and often ignored by patients. The prognosis of CRC is closely related to its early diagnosis. Domestic and foreign studies have found that early diagnosis of CRC can lead to disease cure, with a stage I patient survival rate of 90% at 5 years compared to only 10% in stage IV patients (Brenner et al., 2014). Thus, early detection of colorectal polyps and precancerous lesions and studying the influencing factors are expected to further decrease the morbidity of CRC.

At present, methods for screening CRC and colorectal polyps include fecal DNA detection, fecal immunochemical test (FIT), fecal occult blood test (FOBT), and endoscopic and radiological examinations (Ladabaum et al., 2020; Jain et al., 2022). However, these screening methods have low sensitivity and specificity and issues related to patient compliance. Despite the fact that colonoscopy is the “gold standard” for detecting colorectal polyps, patient compliance is low because it is invasive, expensive, and may cause trauma to the subject, thereby limiting the clinical application of colonoscopy (Chan and Liang, 2022). FOBT and FIT have advantages of being noninvasive and rapid; however, the former has disadvantages of high sampling frequency and low sensitivity (approximately 30–50% for CRC and 10–30% for precancerous adenomas), while the latter has disadvantages of high cost and low sensitivity (approximately 50–60% for CRC and 30% for precancerous adenomas; Ramdzan et al., 2019; Robertson and Selby, 2020). Further, DNA-based bowel cancer screening technology has advantages of being noninvasive and highly sensitive; however, it has a higher false positive rate and more expensive than that of FIT (Sharma, 2020). Therefore, there is an urgent need for an efficient and safe screening method.

Numerous studies have demonstrated that oral microbiota is crucial for identifying and forecasting chronic infectious diseases (Read et al., 2021; Tuominen and Rautava, 2021; Peng et al., 2022). Oral microbiota, as a “sensor” of sub-health states, has important advantages for disease prediction compared to the microbiota in other parts of the human body (Gao et al., 2018). First, the oral cavity serves as the entrance of the digestive tract and the main transportation hub, and several different microorganisms densely colonize the surface of soft and hard tissues of oral cavity. Microorganisms engage in multiple interactions and are closely linked to both health and disease (Tuganbaev et al., 2022). By means of next generation sequencing, researchers found multiple oral pathogens enriched in both colorectal cancerous tissues and feces (Gao et al., 2022; Zhou et al., 2022; Senthakumaran et al., 2023). For example, enrichment of oral biofilm-associated bacteria, *Fusobacterium*, *Gemella*, *Parvimonas*, *Granulicatella*, *Leptotrichia*, *Peptostreptococcus*, *Campylobacter*, *Selenomonas*, *Porphyromonas*, and *Prevotella* in cancer patients compared to adenomatous polyp patients and control patients (Cheng et al., 2020; Gao et al., 2022; Senthakumaran et al., 2023). Second, the oral sample collection is relatively easy, noninvasive, and has high patient compliance. Self-sampling for saliva, dental plaque, and oral mucus samples is possible through simple training of subjects, which can help achieve remote monitoring suitable for long-term follow-up observation (Krahel et al., 2022). In addition, unlike the intestinal microbiota, oral plaque is generally structurally stable (Hall et al., 2017). In samples tested so far, the difference between oral plaque in different disease states is significantly greater than that between individual patients and healthy people (Gao et al., 2022). Until August 2022, a systematic review has identified microbiome-derived biomarkers for early CRC detection which included 28 studies (Zwezerijnen-Jiwa et al., 2023), and only two studies have used oral microbiota as biomarkers (Flemer et al., 2018; Zhang et al., 2020). The overall performance of bacteria-derived biomarkers for the detection of precursor lesions in CRC showed an area under the receiver operating characteristic curve (AUC) ranging from 0.28 to 0.98, a sensitivity ranging from 0.18–1.00 and specificity ranging from 0.39 to 0.97. Notably, the two studies showed high AUC for detection of precursor lesions by using oral microbiota instead of or in conjunction with fecal samples (Flemer et al., 2018; Zhang et al., 2020). These studies were conducted in Western nations, where patients have different genetic and ethnic backgrounds than those in Asian regions, which may have an impact on the microbiota composition (Zackular et al., 2014; Baxter et al., 2016; Eklöf et al., 2017; Bosch et al., 2022; Coker et al., 2022). Furthermore, the two studies employed oral mucosa-based detection of bacterial strains. However, no studies have used saliva samples to predict colorectal polyps using microbial-derived biomarkers.

In this case–control study, the full-length 16S rRNA sequencing was employed to detect the distribution of salivary and fecal microbiota in patients with colorectal polyps and healthy controls. The goal of the study was to clarify the microbial ecology underlying the adenoma-carcinoma sequence and establish the significance of salivary and fecal microbial communities in predicting the presence of colorectal polyps, thereby actively preventing the occurrence and development of CRC, which has rarely been reported in domestic and international studies.

## 2. Materials and methods

### 2.1. Subjects

In this case–control study, we randomly selected patients newly diagnosed with colorectal polyps independently by two expert gastrointestinal pathologists at the Shanghai Fifth People’s Hospital, Fudan University, from May 2021 to November 2022, as the case group (CP group) [conforming to the Rome-IV diagnostic criteria (Drossman, 2016)]. During the same period, the control subjects were family members of patients with colorectal polyps matched by age, sex, body mass index (BMI), dietary habits, oral hygiene habits, and absence of intestinal disease by colonoscopy (HC group). All participants were aged between 18 and 80 years. Participants were excluded if they met the following exclusion criteria: patients who declined to take part in the study or their families, patients who had cognitive issues that made it difficult for them to cooperate with the researchers, patients with previous history of gastrointestinal disease and family history of colorectal polyps in a first-degree relative, patients with any of the following diseases (autoimmune diseases such as systemic lupus erythematosus and ankylosing spondylitis; organ failure; cachexia; infectious diseases; cardiovascular and respiratory diseases), patients who were pregnant or lactating; patients with BMI < 18.5 kg/m^2^ or BMI > 32 kg/m^2^; patients who had suffered from oral diseases, patients who had received antibiotics, probiotics, microbioactive bacterial preparations, or berberine within the preceding three months, and patients with concurrent major disorders and an alcohol or drug abuse history.

This study was authorized by the Shanghai Fifth People’s Hospital Ethics Committee, Fudan University [(2021) 127], and it was carried out in accordance with the Declaration of Helsinki of the World Medical Association. An informed consent form was signed by all enrolled participants.

### 2.2. Methods

#### 2.2.1. Questionnaire survey

We designed a questionnaire based on the World Health Organization’s (WHO) “Oral Health Survey: Basic Methods” (the 5th edition) (Yeung, 2014) that included items about age, sex, education level, occupation, height and weight, smoking habit, oral hygiene behavior, general health status, and the size, site, number (single and multiple), and pathological type of polyps, among other things. On-site, trained researchers distributed the questionnaires to the participants, gave them instructions for filling them out, and collected the completed forms.

#### 2.2.2. Collection of salivary and fecal samples and methods for detection

Between 8:00 and 11:00 in the morning, salivary samples were taken from each patient. The participants were told not to consume any food or beverages, smoke, or practice any oral hygiene procedures 2 h before sampling. The participants gargled with deionized water and collected unstimulated saliva (at least 5 mL) in a plastic cup (Ebersole et al., 2013). If blood was present in the saliva, it was discarded and collected again. The collected salivary samples were immediately transferred to a centrifuge tube and centrifuged at 4°C for 10 min at a speed of 7,000 r/min. The supernatant was collected and divided into Eppendorf tubes, which were immediately stored at −80°C. Repeated freeze-thawing of salivary samples were avoided during the study.

For all subjects, approximately 3–5 g fresh fecal specimens from the middle section were collected using a special fecal kit (Shanghai Personalbio Technology Co., Ltd., Shanghai, China), immediately frozen at −20°C, stored in a dry ice box and transported to the laboratory, and stored at −80°C until further analysis.

Full-length 16S rRNA sequencing was used to investigate salivary and fecal samples, and the distribution of the microbiota in samples from the CP and HC groups was detected. Following the manufacturer’s instructions, total genomic DNA samples were extracted using the Mag-Bind Blood & Tissue DNA HDQ 96 Kit (M6399-01, Omega, Inc., United States). NanoDrop NC2000 spectrophotometer (Thermo Fisher Scientific, Waltham, Massachusetts, USA) and agarose gel electrophoresis were used to quantify the quantity and quality of the extracted DNA, respectively. The forward primer 27F (5’-AGAGTTTGATCMTGGCTCAG-3′) and the reverse primer 1492R (5’-ACCTTGTTACGACTT-3′) were used in PCR to amplify virtually full-length bacterial 16S rRNA genes. The collected DNA was amplified using a two-stage PCR process, with the second PCR step including sample-specific 16-bp barcodes into the forward and reverse primers for multiplex sequencing. Both the two steps of the PCR components (New England Biolabs, Ipswich, MA, USA) contained 5 μL of Q5 reaction buffer (5×), 5 μL of Q5 High-Fidelity GC buffer (5×), 0.25 μL of Q5 High-Fidelity DNA Polymerase (5 U/μl), 2 μL (2.5 mM) of dNTPs, 1 μL (10 μM) of each Forward and Reverse primer, 2 μL of DNA Template, and 8.75 μL of ddH_2_O. Thermal cycling consisted of initial denaturation at 98°C for 2 min, followed by 25/10 cycles (for first and second amplification step, respectively) consisting of denaturation at 98°C for 30 s, annealing at 55°C for 30 s, and extension at 72°C for 90 s, with a final extension of 5 min at 72°C. The PicoGreen dsDNA Assay Kit (Invitrogen, Carlsbad, CA, USA) was used to measure the quantity of PCR amplicons after they had been purified using Agencourt AMPure Beads (Beckman Coulter, Indianapolis, IN). At Shanghai Personal Biotechnology Co., Ltd. (Shanghai, China), amplicons were pooled in identical proportions following the individual quantification phase and Single Molecule Real Time (SMRT) sequencing technology was carried out utilizing the PacBio Sequel platform. PacBio circular consensus sequencing (CCS) reads were produced using multiple alignments of sub-reads to reduce the sequencing error rate. With CCS, a circular DNA template that has been ligated is read by the DNA polymerase several times, effectively producing a consensus sequence from numerous readings of a single molecule. Initial processing of raw sequences took place via the PacBio SMRT Link portal (version 5.0.1.9585). Sequences were filtered with at least three passes and at least 99% predicted accuracy (minfullpass = 3, minPredicted Accuracy = 99). The level below which a CCS is regarded as noise is defined as the projected accuracy of 99%. The sequences larger than 2,000 bp were then removed from the files created by the PacBio platform using amplicon size trimming.

QIIME2 was used to carry out microbiome bioinformatics with a minor modification in accordance with the official tutorials.^1^ In brief, primers were cut with the cutadapt plugin after raw sequence data were demultiplexed using the demux plugin. Then, using the Vsearch plugen’s fastq_mergepairs, fastq_filter, and derep_fullength functions, sequences were combined, quality filtered, and dereplicated. Following a 98% clustering of all the distinct sequences (using cluster_size), chimeras were then eliminated (using uchime_denovo). In order to create Operational Taxonomic Unit (OTU) representive sequences and an OTU table, the non-chimera sequences were ultimately re-clustered at a 97% level. Using fasttree2, non-singleton OTUs were aligned with mafft and used to build a phylogeny. Based on the Silva database, the RDP Classifier was used to classify representative sequences of each OTU into various taxonomic groups.

### 2.3. Statistical analysis

The SPSS version 25 (IBM Corp., Armonk, NY, United States), QIIME2, and R packages (v3.2.0) were used to conduct the statistical analyses. Patients without sufficient data were disqualified. Continuous variables with or without a normal distribution were shown as median (interquartile range) or mean ± standard deviation. Depending on whether a normal distribution existed or not, the independent samples t-test or Kruskal-Wallis test was used to compare the groups. Intergroup comparisons were done using the *χ*^2^ test or Kruskal-Wallis test, and count data were presented as percentages or ratios. Statistical significance was defined as a *p*-value <0.05. Sequence data analyses were performed using the QIIME2 and R packages (v3.2.0). Using the OTU table in QIIME2, the alpha diversity indices—including the Chao1 richness estimator, Good’s coverage, Shannon diversity index, and Simpson index—were calculated and displayed as box plots. Using unweighted and weighted UniFrac distance metrics and principal coordinate analysis (PCoA), beta diversity analysis was carried out to assess the structural variation in microbial communities across samples. The significance of differences in microbiota structure among the groups was assessed by permutational multivariate analysis of variance (PERMANOVA) using QIIME2. The default parameters of linear discriminant analysis (LDA) effect size (LEfSe) were used to identify differentially abundant taxa across groups. The screening value for the LDA Score is 2. To distinguish samples from various groups, random forest analysis was used with QIIME2’s default settings. Automated hyperparameter optimization and sample prediction employed nested stratified 10-fold cross-validation. The differential species obtained by the LEfSe analysis and top 30 species of importance obtained by the random forest analysis were used as diagnostic marker species. A diagnostic model based on these bacterial markers was used to classify colorectal polyps. A receiver operating characteristic (ROC) curve was generated to calculate the AUC value. This evaluated the effectiveness of the diagnostic model and assessed whether these species could be used as potential diagnostic markers using the R software. What’s more, Spearman’s correlation analysis was performed on these differential metabolites and gut microbiota.

The NCBI Sequence Read Archive (The BioProject number PRJNA957055) provided the raw sequences shown in this study.

## 3. Results

### 3.1. Study population

The current study involved 55 participants in total, 33 of whom had colorectal polyps. Patients with colorectal polyps and controls were matched by age, sex, BMI, education level, smoking history, frequency of tooth brushing per day, and frequency of dental visits (*p* > 0.05). The demographic data of the participants are presented in Table 1.

**Table 1:** Demographic characteristics of the subjects.

Characteristics		Control group	CP group	*p*-value
		(*n* = 22)	(*n* = 33)	
Age (mean ± SD)		61.45 ± 6.93	59.12 ± 9.82	0.339^a^
BMI (mean ± SD), kg/m^2^		23.09 ± 2.19	23.74 ± 3.21	0.376^a^
Sex				0.178^b^
	Male	9 (40.9)	20 (60.6)	
	Female	13 (59.1)	13 (39.4)	
Education level				0.853^b^
	Illiteracy	1 (4.5)	1 (3.0)	
	Junior school	5 (22.7)	10 (30.3)	
	Junior high school	11 (50.0)	17 (51.5)	
	High school or above	5 (22.7)	5 (15.2)	
Vocation				0.623^b^
	Retiree	11 (50.0)	18 (54.5)	
	Farmer	2 (9.1)	1 (3.0)	
	Worker	9 (40.9)	14 (42.4)	
Diabetes				
	Yes	2 (9.1)	3 (9.1)	1.000^b^
	No	20 (90.9)	30 (90.9)	
Hypertension				0.800^b^
	Yes	6 (27.3)	8 (24.2)	
	No	16 (72.7)	25 (75.8)	
Smoking status				0.826^b^
	Never	17 (77.3)	23 (69.7)	
	Ex	2 (9.1)	4 (12.1)	
	Current	3 (13.6)	6 (18.2)	
Alcohol consumption				0.181^b^
	Never	13 (59.1)	25 (75.8)	
	Ex	1 (4.5)	3 (9.1)	
	Current	8 (36.4)	5 (15.2)	
Meat-eating frequency				0.565^b^
	1–2 times/week	6 (27.3)	12 (36.4)	
	>2 times/week	16 (72.7)	21 (63.6)	
Defecation frequency				0.724^b^
	1–2 times/week	2 (9.1)	4 (12.1)	
	1–2 times/day	20 (90.9)	29 (87.9)	
Frequency of tooth brushing				0.767^b^
	< 2 times/day	4 (18.2)	5 (15.2)	
	≥ 2 times/day	18 (81.8)	28 (84.8)	
Frequency of tooth flossing				0.604^b^
	Not every day	16 (72.7)	26 (78.8)	
	Every day	6 (27.3)	7 (21.2)	
Frequency of dental visits				0.660^b^
	≤ 1 time/year	12 (54.5)	16 (48.5)	
	> 1 time/year	10 (45.5)	17 (51.5)	
Exercise				0.889^b^
	Never	10 (45.5)	16 (48.5)	
	Occasionally	8 (36.4)	10 (30.3)	
	Frequently	4 (18.2)	7 (21.2)	
Polyp pathology				
	Non-adenomatous polyps	–	12 (36.4)	
	Adenomatous polyps	–	21 (63.6)	
Polyp position				
	Rectum	–	6 (18.2)	
	Sigmoid colon	–	12 (36.4)	
	Descending colon	–	7 (21.2)	
	Transverse colon	–	5 (15.2)	
	Ascending colon	–	5 (9.1)	
Polyp number				
	Single	–	15 (45.5)	
	More than 2	–	18 (54.5)	
Polyp size (cm)		–	1.12 ± 0.07	

*P*, significance of differences between healthy controls and patients with colorectal polyp.

a Independent samples *t*-test.

b *χ*^2^-test.

c Kruskal–Wallis test.

The kappa coefficient of the intra-examiner and inter-examiner agreement were 0.89 and 0.85, respectively (*p* < 0.001), indicating high reliability and satisfactory agreement.

### 3.2. Alpha diversity in salivary and fecal samples

**Figure 1:**
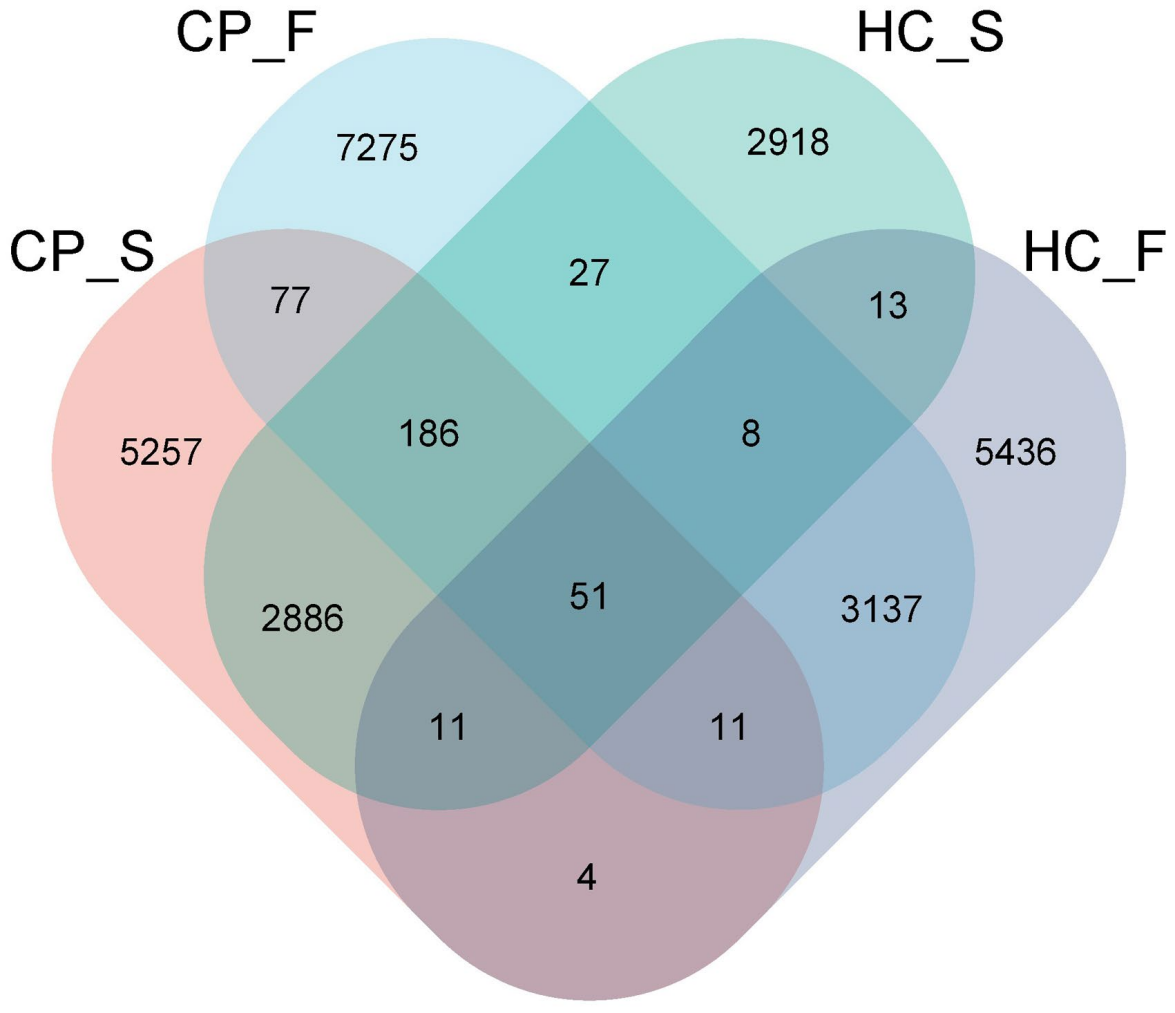
Venn diagram displaying number of the overlapping OTUs among the four study groups. In the figure, each patch represents a group, the overlapping area between the blocks indicates the OTUs common to the corresponding group, and the number of each block indicates the number of OTUs contained in the block. CP_S: salivary samples of colorectal polyp patients; CP_F: fecal samples of colorectal polyp patients; HC_S: salivary samples of healthy controls; HC_F: fecal samples of healthy controls.

For all samples, the sequence length distribution range was 32–3,074 bp, average sequence length was 1,455 bp, and average number of OTUs per sample was 580 ± 28. The average number of microbial taxa at each level in all samples was 6 phyla, 12 classes, 18 orders, 28 families, 37 genera, and 41 species. The number of OTUs in the HC_S (controls-salivary sample), HC_F (controls-fecal sample), CP_S (polyps-salivary sample), and CP_F (polyps-fecal sample) groups were 5,257, 7,275, 2,918, and 5,436, respectively, of which 51 were shared among the four groups (Figure 1).

**Figure 2:**
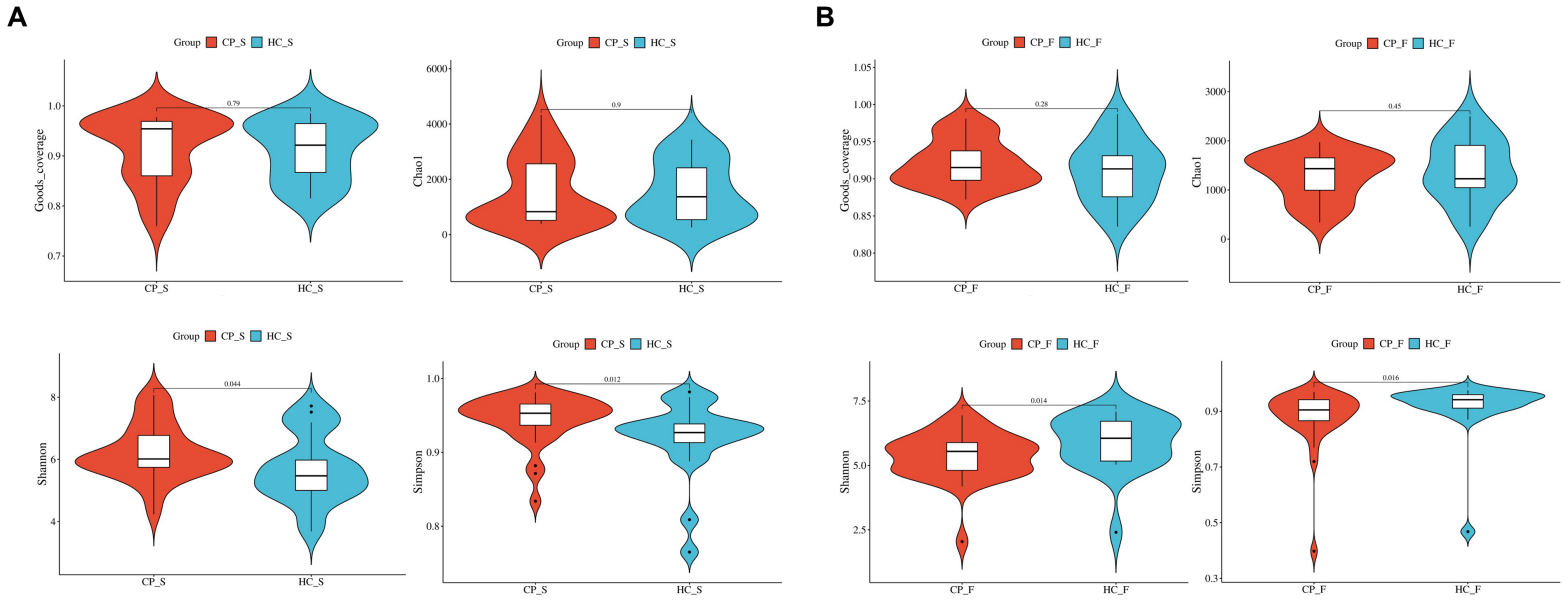
Comparison of α-diversity of **(A)** salivary microbiota and **(B)** fecal microbiota between patients with colorectal polyps and healthy controls shows that Shannon diversity and Simpson indices of salivary samples from patients with colorectal polyp were significantly higher than controls, but completely reversed in fecal samples. The abscissa is the group label, and the ordinate is the value of the corresponding alpha diversity index. In the box plot, the symbols mean the following: the upper and lower end of the box, and the upper and lower Interquartile range (IQR); Median, median; Upper and lower edges, maximum and minimum; The points outside the upper and lower edges represent outliers. The numbers in the figure are the *p*-values of the Kruskal-Wallis test. CP_S: salivary samples of colorectal polyp patients; HC_S: salivary samples of healthy controls; CP_F: fecal samples of colorectal polyp patients; HC_F: fecal samples of healthy controls.

Shannon diversity and Simpson indices of salivary samples from patients with colorectal polyp were increased compared to the controls (*p* = 0.044 and *p* = 0.013, respectively); however, there was no significant difference between Good’s coverage and Chao1 (*p* = 0.783 and *p* = 0.894, respectively), as shown in Figure 2A. However, fecal samples from the polyp group’s Shannon diversity and Simpson indices were significantly lower than those from the control group (*p* = 0.015 and *p* = 0.017, respectively; Figure 2B).

### 3.3. Salivary and fecal microbiota structures in the colorectal polyp and control groups

**Figure 3:**
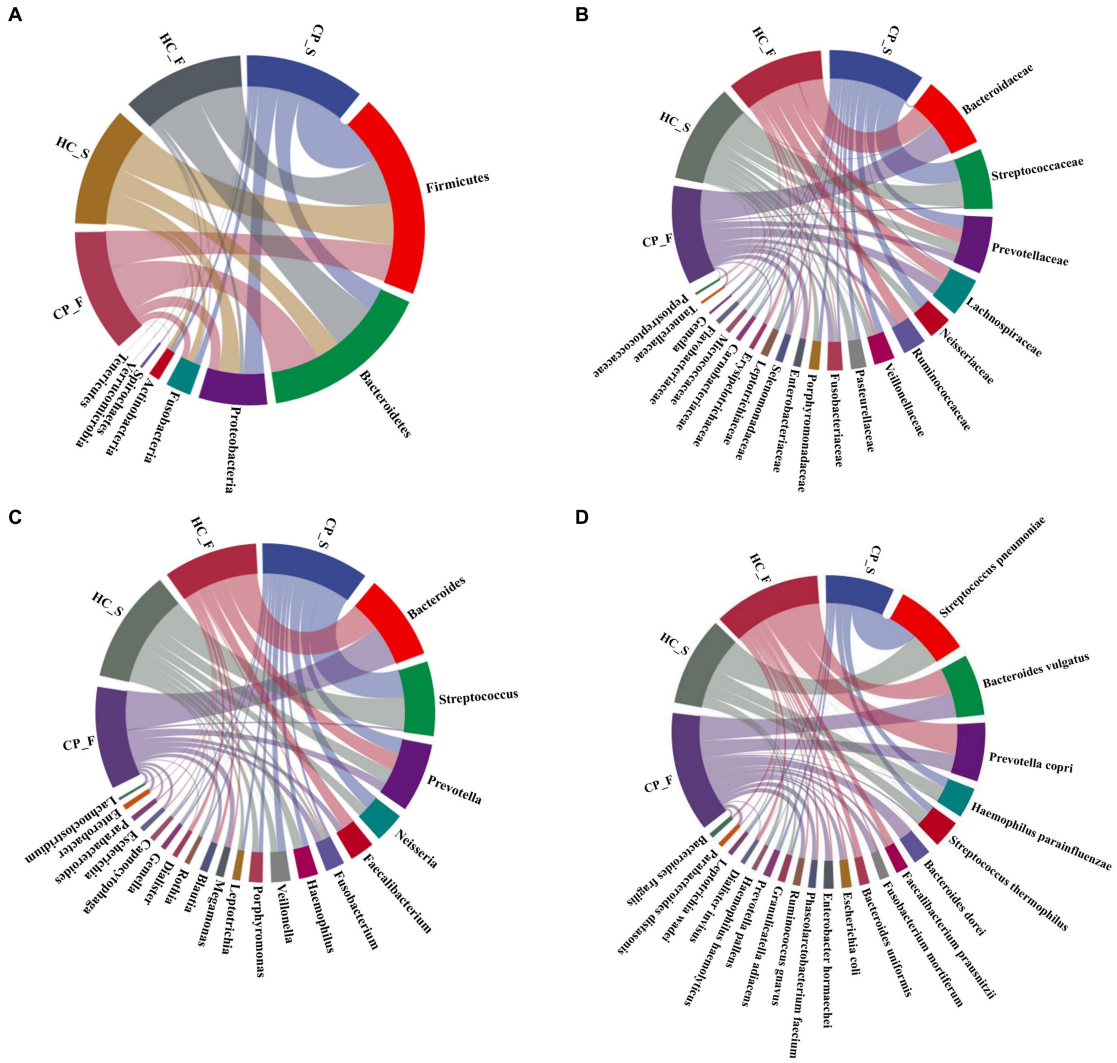
Composition and distribution of the main members in microbiota from saliva and feces of the polyp and control groups [**(A)**: at the phylum level; **(B)**: top 20 at the family level; **(C)**: top 20 at the genus level; **(D)**: top 20 at the species level]. In the chord diagram, lines are used to show the distribution characteristics of microorganisms at different levels in the four groups, and colors and line thicknesses are used to show different types of connections and strengths. CP_F: fecal samples of colorectal polyp patients; HC_S: salivary samples of healthy controls; HC_F: fecal samples of healthy controls; CP_S: salivary samples of colorectal polyp patients.

**Figure 4:**
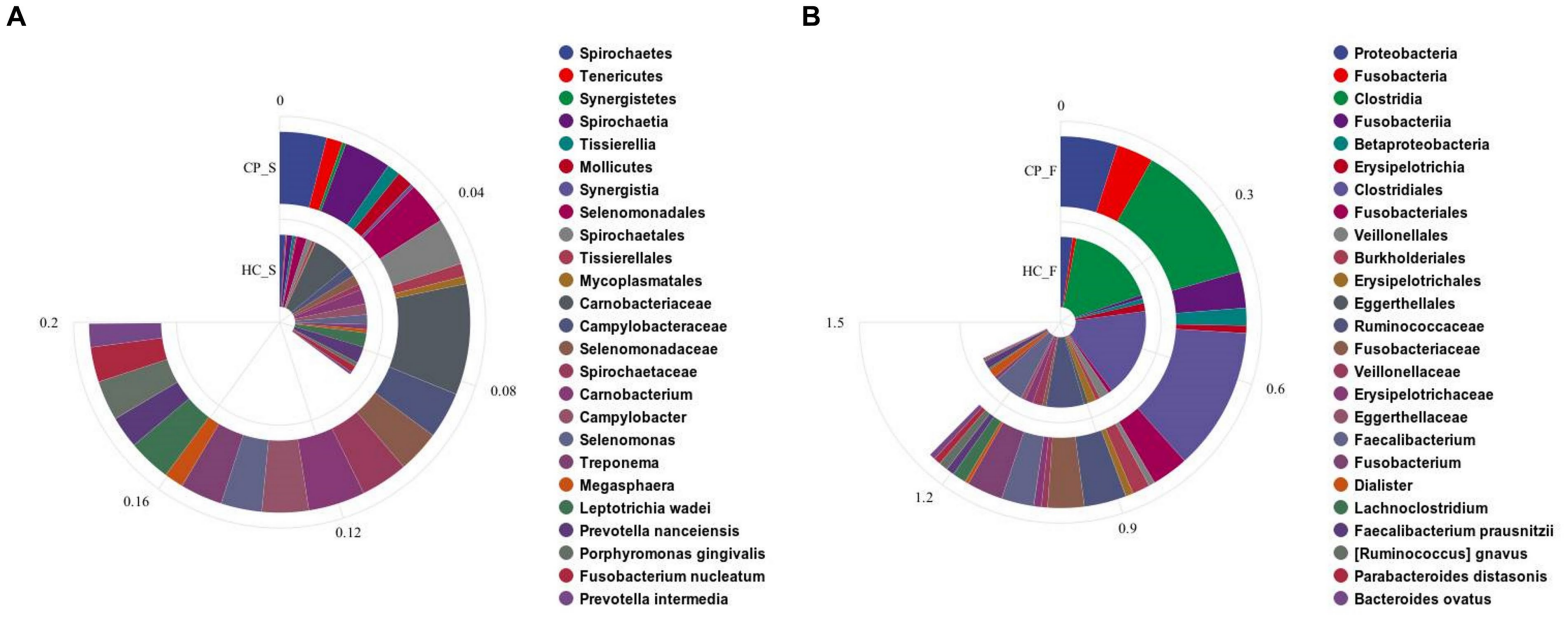
Bar charts showing significant differences in the abundance of **(A)** salivary microbiota and **(B)** fecal microbiota at the levels of phylum, class, order, family, genus, and species by using Kruskal–Wallis tests between the colorectal polyp and the control group. CP_S: salivary samples of colorectal polyp patients; HC_S: salivary samples of healthy controls; CP_F: fecal samples of colorectal polyp patients; HC_F: fecal samples of healthy controls.

The composition and distribution of the top 20 at the phylum, family, genus, and species levels are shown in Figures 3A–D. Figures 4A,B illustrated the differences in the fecal and salivary microbiota between the CP and HC groups at each level of classification.

Compared with the controls, salivary microbiota of the CP group showed an increased abundance of *Spirochaetes*, *Tenericutes*, and *Synergistetes* at the phylum level; *Carnobacterium*, *Campylobacter*, *Selenomonas*, *Treponema*, and *Megasphaera* at the genus level; and *Leptotrichia wadei*, *Porphyromonas gingivalis*, *Fusobacterium nucleatum*, and *Prevotella intermedia* at the species level (*p* < 0.05). The abundance of *Prevotella nanceiensis* decreased in patients with colorectal polyps (*p* < 0.05).

Fecal samples of the CP group showed increased abundance of the phylum *Proteobacteria* and *Fusobacteria*, genus *Fusobacterium* and *Lachnoclostridium*, species *Ruminococcus gnavus*, *Parabacteroides distasonis*, and *Bacteroides ovatus*, compared with those of the HC group (*p* < 0.05), whereas the genus *Faecalibacterium* and *Dialister* and the species *Faecalibacterium prausnitzii* had decreased abundance (*p* < 0.05); in addition, although there was a downward trend in the phyla Firmicutes and Tenericutes, the difference was not statistically significant. (*p* = 0.069 and *p* = 0.058, respectively; Figure 4B).

### 3.4. Beta diversity in saliva and fecal samples

**Figure 5:**
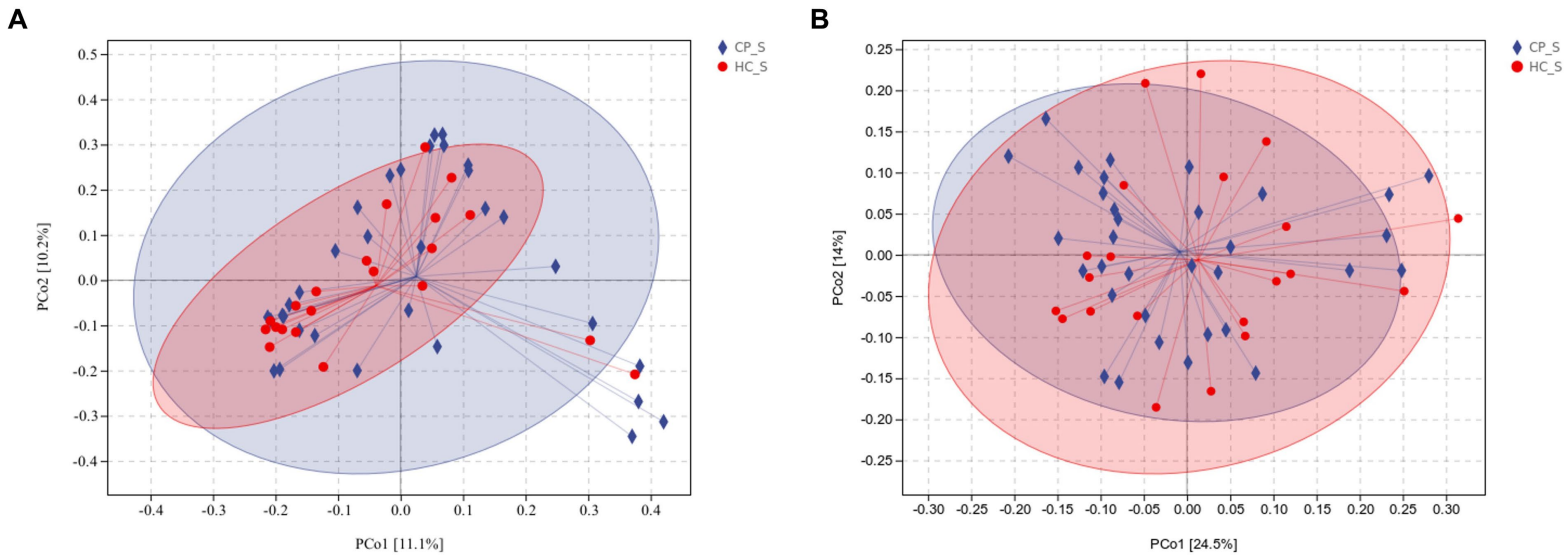
**(A)** Unweighted and **(B)** weighted UniFrac-based PCoA of salivary microbiota shows significant distinction in β-diversity between the CP and HC groups. Each point in the plot represents a sample, and different colored points indicate different groups. The percentages in parentheses on the axes represent the fraction of the sample variance data (distance matrix) that can be explained by the corresponding axes. A 95% confidence ellipse is drawn. CP_S: salivary samples of colorectal polyp patients; HC_S: salivary samples of healthy controls.

**Figure 6:**
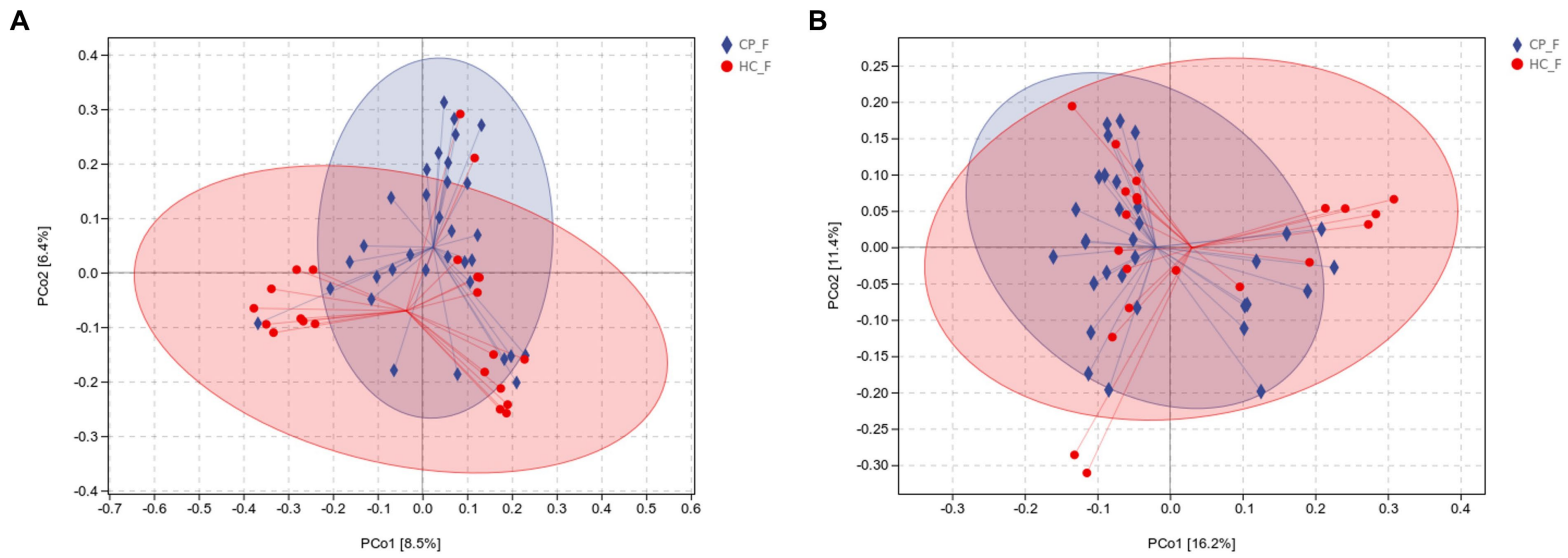
**(A)** Unweighted and **(B)** weighted UniFrac-based PCoA of fecal microbiota shows significant distinction in β-diversity between the CP and HC groups. Each point in the plot represents a sample, and different colored points indicate different groups. The percentages in parentheses on the axes represent the fraction of the sample variance data (distance matrix) that can be explained by the corresponding axes. A 95% confidence ellipse is drawn. CP_F: fecal samples of colorectal polyp patients; HC_F: fecal samples of healthy controls.

Beta diversity was calculated using unweighted and weighted UniFrac distance metrics and PCoA to assess the microbiota distribution of different samples (Figures 5A,B, Figures 6A,B). The results showed significant differences in the microbiota distribution of salivary and fecal samples between the CP and HC groups using PERMANOVA (saliva: *p* = 0.028; feces: *p* = 0.022).

### 3.5. Heatplot of correlation between OTUs detected in saliva and fecal samples

**Figure 7:**
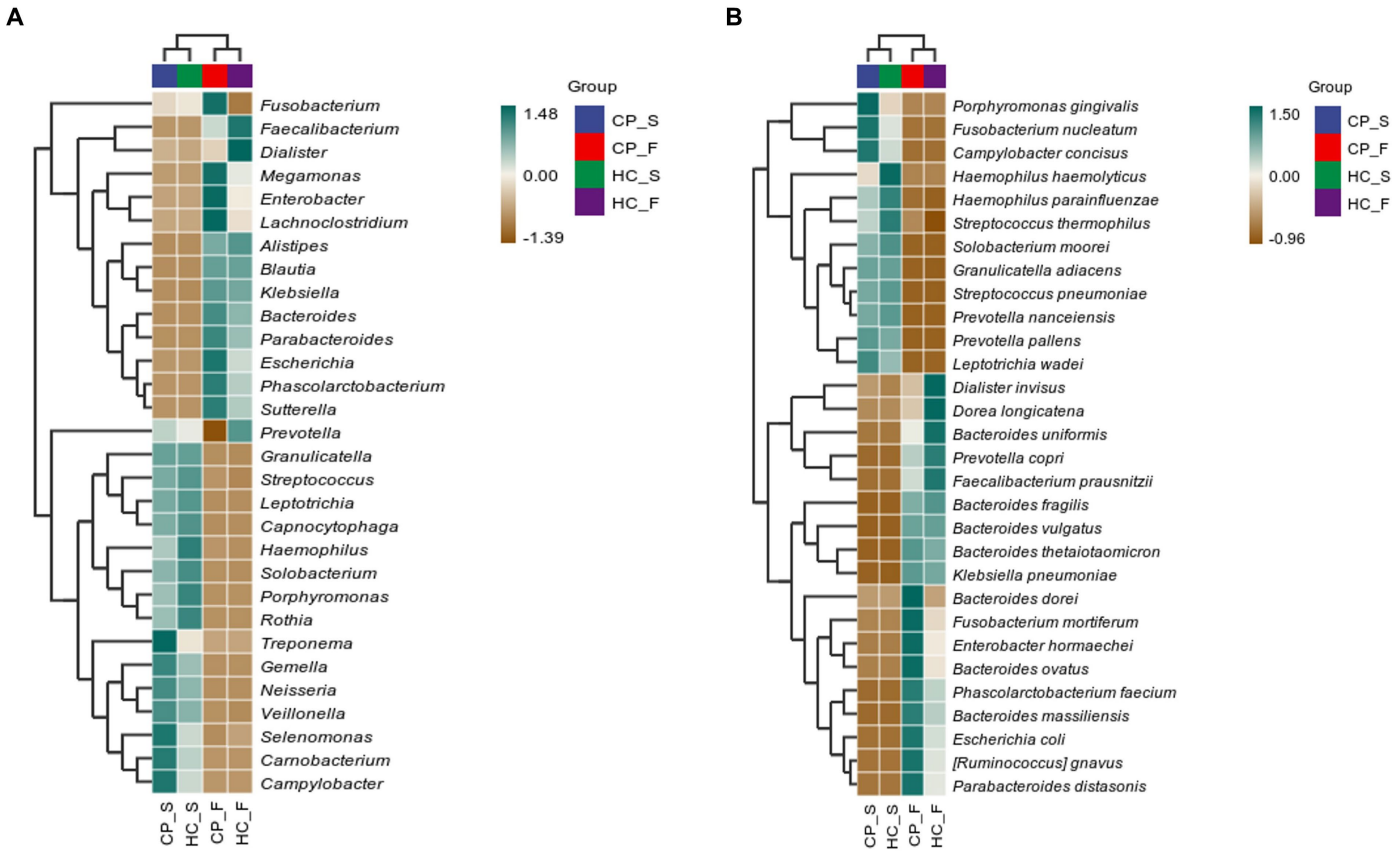
Correlation heatplots of **(A)** the top 20 taxa at genus level and **(B)** the top 20 taxa at species level detected in salivary and fecal samples from patients with colorectal polyps and healthy controls. CP_S: salivary samples of colorectal polyp patients; CP_F: fecal samples of colorectal polyp patients; HC_S: salivary samples of healthy controls; HC_F: fecal samples of healthy controls.

To further compare microbiota differences, we constructed heatplots using data of the top 20 at the genus and species levels (Figures 7A,B). In patients with colorectal polyps, *P. gingivalis*, *F. nucleatum*, and *Campylobacter concisus* were more abundant in the saliva, and *Bacteroides dorei*, *Fusobacterium mortiferum*, *Enterobacter hormaechei*, *B. ovatus*, *Phascolarctobacterium faecium*, *Bacteroides massiliensis*, *Escherichia coli*, *R. gnavus*, and *P. distasonis* were enriched in the feces.

### 3.6. LEfSe analysis of salivary and fecal samples

**Figure 8:**
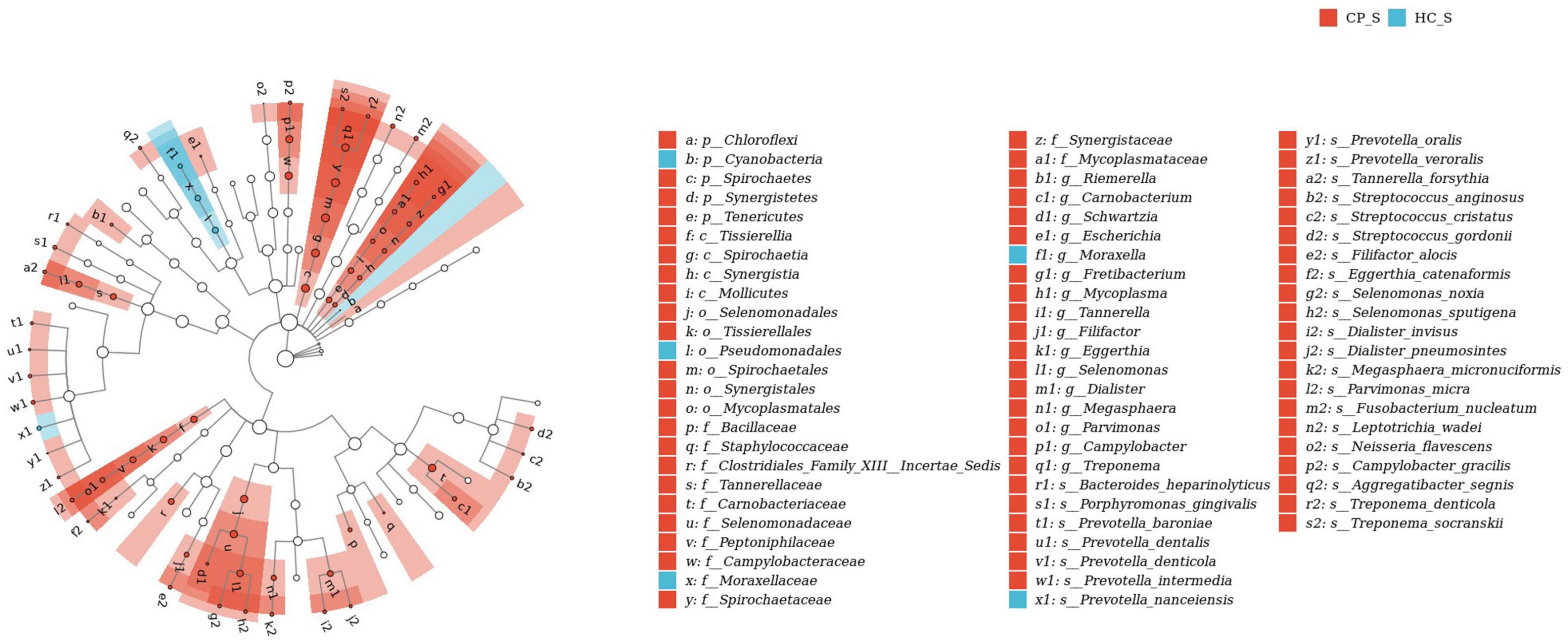
LEfSe biomarker analysis of salivary samples from patients with colorectal polyps shows taxonomic rank relationships of the main taxa from phylum to species (inner ring to outer ring). The taxonomic cladogram shows the taxonomic hierarchical relationships of the main taxa from phylum to genus, from inner to outer circles, in the sample community. Node size corresponds to the average relative abundance of that taxon; Hollow nodes represent taxa that do not differ significantly between groups, while nodes in other colors (e.g., blue and red) indicate that these taxa exhibit significant group differences and are more abundant in the group samples represented by that color. Letters identify the names of taxa that differ significantly between groups. CP_S: salivary samples of colorectal polyp patients; HC_S: salivary samples of healthy controls.

**Figure 9:**
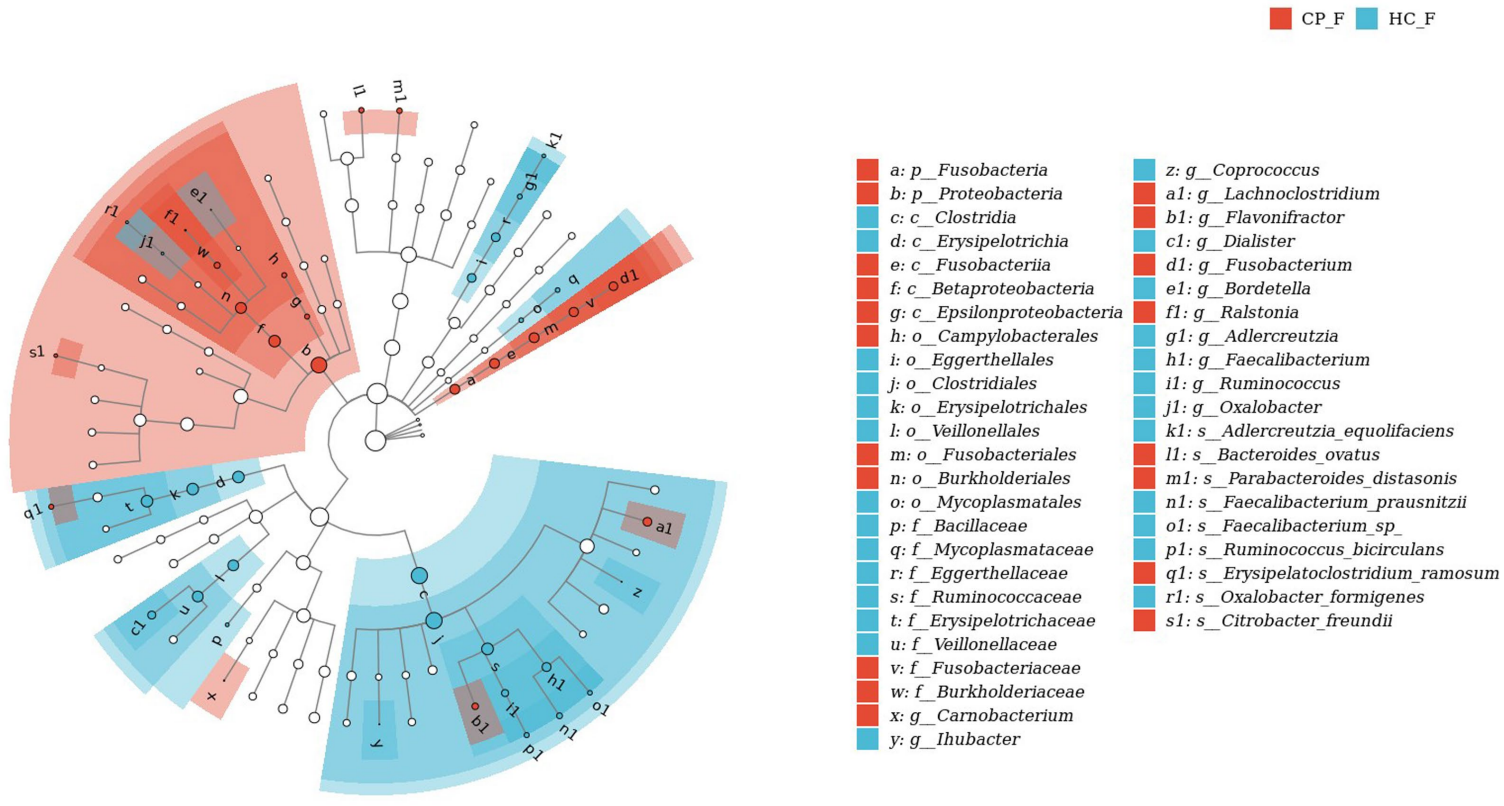
LEfSe biomarker analysis of fecal samples from patients with colorectal polyps shows taxonomic rank relationships of the main taxa from phylum to species (inner ring to outer ring). The taxonomic cladogram shows the taxonomic hierarchical relationships of the main taxa from phylum to genus, from inner to outer circles, in the sample community. Node size corresponds to the average relative abundance of that taxon; Hollow nodes represent taxa that do not differ significantly between groups, while nodes in other colors (e.g., blue and red) indicate that these taxa exhibit significant group differences and are more abundant in the group samples represented by that color. Letters identify the names of taxa that differ significantly between groups. CP_F: fecal samples of colorectal polyp patients; HC_F: fecal samples of healthy controls.

LefSe analysis was used to estimate the differences in abundance of the identified taxa between the healthy and case groups. *P. gingivalis*, *L. wadei*, *F. nucleatum*, *P. intermedia*, and *Megasphaera micronuciformis* were identified as the major contributors to the salivary microbiota of patients with colorectal polyps (the LDA score > 2; Figure 8). The LDA scores indicated relatively higher abundance of *R. gnavus*, *B. ovatus*, *P. distasonis*, *Citrobacter freundii*, and *Clostridium symbiosum* in the fecal microbiota of the CP group than that of the HC group (the LDA score > 2; Figure 9). In contrast, *Prevotella nanceiensis*, *F. prausnitzii*, *Adlercreutzia equolifaciens*, and *Ruminococcus bicirculans* were enriched in both salivary and fecal microbiota of the controls (Figures 8, 9, red bars).

### 3.7. Identification and validation of salivary and fecal microbial OTU-based biomarkers for colorectal polyp

**Figure 10:**
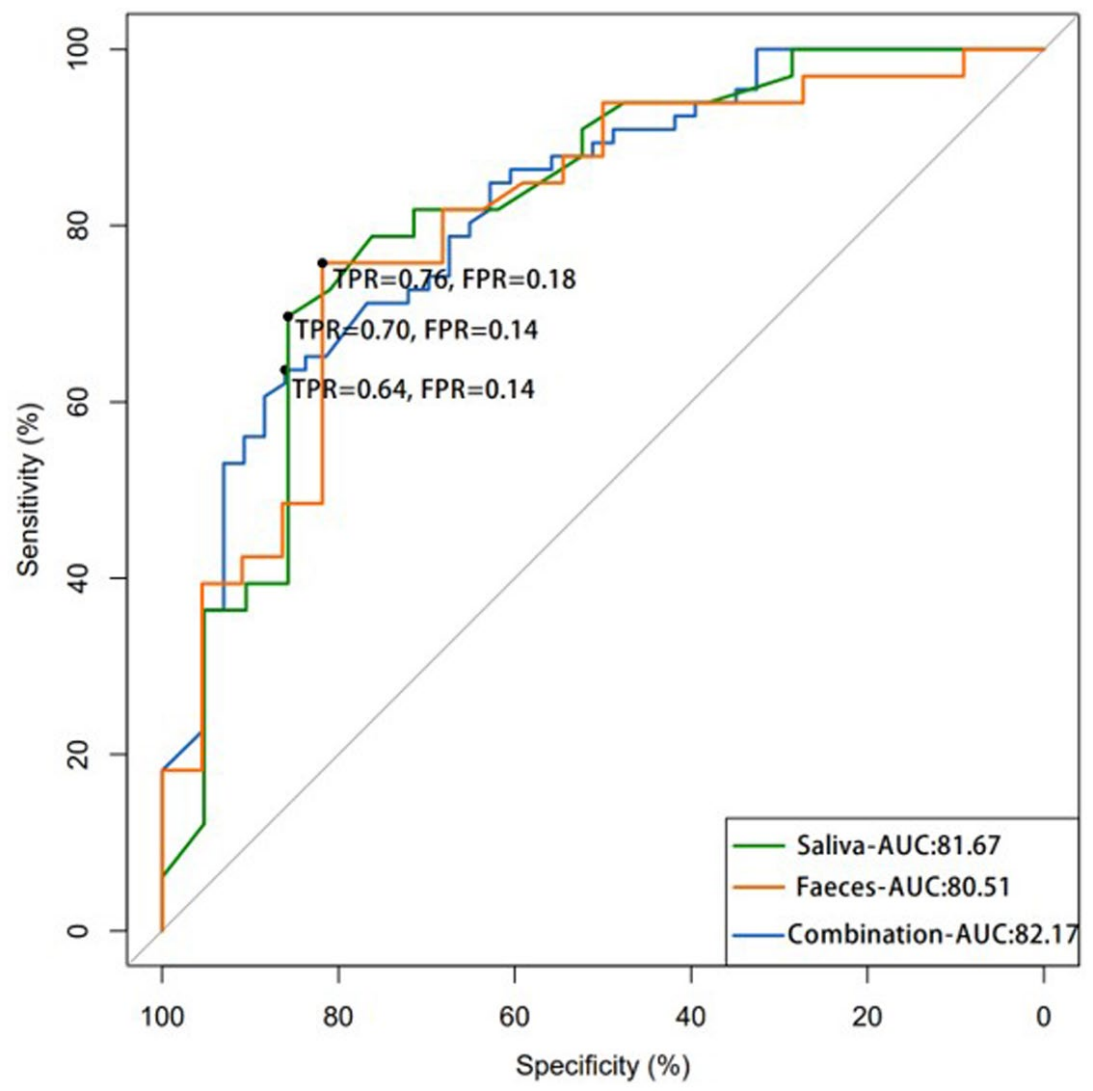
The ROC curve demonstrates the efficacy of using salivary and fecal microbiota as potential tool for detecting colorectal polyps, showing that the combination of both samples microbiota may result in more accurate detection. (AUC: area under the curve; FPR: false-positive rate; TPR: true-positive rate).

The significance of the salivary and fecal microbiota as biomarkers of colorectal polyps was evaluated. ROC curves were used to analyze the degree of correlation between colorectal polyps and identified taxa. This model identified 32 salivary microbiota species that distinguished individuals with colorectal polyps from healthy controls. The sensitivity and specificity of detection were 76% and 81.67%, respectively (AUC: 0.8167) (Figure 10 and Online Supplementary Table 1). The top 10 species of importance were *Parvimonas micra*, *Streptococcus gordonii*, *Streptococcus anginosus*, *Prevotella denticola*, *Tannerella forsythia*, *P. nanceiensis*, *P. intermedia*, *L. wadei*, *L.* sp. oral taxon 498, and *F. nucleatum*.

The sensitivity of our model in detecting 11 microbiota species in fecal samples of individuals with polyps was 70% with an AUC of 0.8051 (Figure 10 and Online Supplementary Table 2). The top 10 species of importance were, *F. prausnitzii*, *P. distasonis*, *R. gnavus*, *B. ovatus*, *Clostridium symbiosum*, *A. equolifaciens*, *Erysipelatoclostridium ramosum*, *R. bicirculans*, and *Oxalobacter formigenes*. A combination of 30 salivary and fecal microbiota datasets improved model sensitivity to 64%, with an AUC of 0.8217 for the detection of polyps (Figure 10 and Online Supplementary Table 3). The top 10 species of importance were *Streptococcus thermophilus*, *S. gordonii*, *S. anginosus*, *P. denticola*, *Haemophilus parainfluenzae*, *T. forsythia*, *Eubacterium nodatum*, *Selenomonas sputigena*, and *P. micra*. The AUC values were highest for the combination test. Thus, this analysis indicated the significance and reliability of the abundance data of *Streptococcus*, *Prevotella*, *Tannerella*, and *Faecalibacterium* to identify samples from patients with polyps.

### 3.8. Association network of salivary and fecal microbiota in patients with colorectal polyps

**Figure 11:**
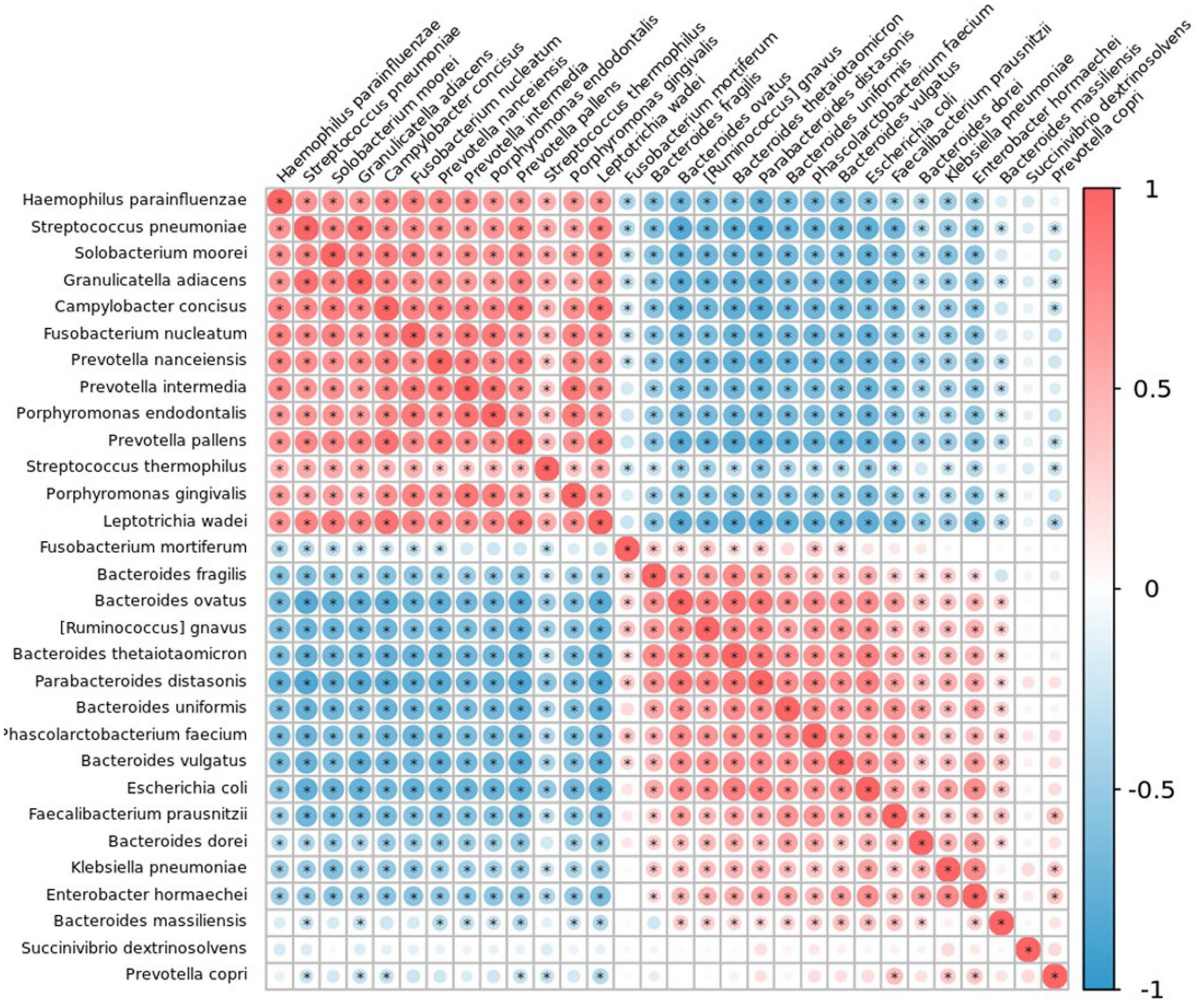
Correlation plot of the top 30 taxa at species level in patients with colorectal polyps using Spearman’s correlation. Correlations with an adjusted *p*-value less than 0.05 are displayed with a dot inside the circle. Blue indicates a negative correlation, red indicates a positive correlation, with darker colors showing stronger correlations.

The microbial relationship between salivary and fecal systems was determined using Spearman’s correlation to analyze statistical differences in the species abundance (Figure 11 and Online Supplementary Table 4). *F. nucleatum* was negatively correlated with *F. prausnitzii* (*R* = −0.265; *p* = 0.032) and *P. distasonis* (*R* = −0.296; *p* = 0.016). *L. wadei* correlated with *P. gingivalis* (*R* = 0.437; *p* < 0.001) and *F. nucleatum* (*R* = 0.342; *p* = 0.005).

## 4. Discussion

The pathogenesis of colorectal polyps is multifactorial and complex, which involves molecular genetics and histopathological changes (Tse et al., 2021). Studies have confirmed that family history, ulcerative colitis, diets high in fat and low in fiber are risk factors for colorectal polyps (Haque et al., 2014; Sninsky et al., 2022). With the continuous development of molecular biotechnology, in-depth metagenomic studies, and wide application of second-generation sequencing technology, the role of intestinal colonizing microbiota in the pathogenesis of colorectal polyps has become a clinical research focus in recent years (Avelar-Barragan et al., 2022; Bosch et al., 2022; Xu et al., 2022; Zhong et al., 2022). Although metagenomic sequencing allows high-resolution species identification, it is cost prohibitive for many researchers (Joseph and Pe'Er, 2021). At present, most studies on the microbiota of patients with colorectal polyps have sequenced only some areas, such as V3, V4 and V5, resulting in severe underestimation of the abundance of microorganisms of interest (Liu et al., 2020; Wei et al., 2020; Zhang et al., 2020). Different variable regions have been reported to have large deviations in species classification ability; the V4 region had the worst ability to distinguish, and the V1-V9 region could realize all sequence annotations for specific species (Singer et al., 2016; Johnson et al., 2019). Therefore, full-length 16S rRNA gene sequencing was performed. Long-read sequences could contain more information on genomic structural variation, thus providing better species resolution and more accurate restoration of the species community structure.

In the present research, we observed significant differences in the salivary and fecal bacteria between the CP and HC groups. Association between fecal microbiota and colorectal polyps have already been reported in many studies (Brim et al., 2013; Hale et al., 2017; Kim et al., 2020; Chen et al., 2021; Coker et al., 2022; Senthakumaran et al., 2023), but a few studies have been conducted on the association between oral microbiota and colorectal polyps (Flemer et al., 2018; Zhang et al., 2022). Our study confirmed that compared with those of controls, Shannon and Simpson indices of salivary samples from the CP group were higher along with increased abundance of some oral pathogens, including *L. wadei*, *P. gingivalis*, *F. nucleatum*, and *P. intermedia*, which is consistent with the findings of Flemer et al. (2018) and Zhang et al. (2022).Other studies have revealed increased abundance of oral microbiota species in fecal samples of patients with colonic adenoma, including *Corynebacterium*, *Actinomyces*, *Porphyromonas*, *Haemophilus*, and *Mogibacterium*, comparing to healthy controls (Flemer et al., 2017; Hale et al., 2017; Liang et al., 2017). These results were not observed in the present study, which could have been explained by inconsistencies resulting from variations in the study subjects, salivary and fecal collection and preservation methods (Hale et al., 2015; Sinha et al., 2016), library preparation (Jones et al., 2015), or sequencing platforms and databases (Hamady and Knight, 2009).

*P. gingivalis* and *F. nucleatum* are important pathogens in periodontitis that can invade human epithelial cells and progress to colorectal adenomas via multiple potential mechanisms (Kostic et al., 2013; Hashemi et al., 2019; Reitano et al., 2021; Wang et al., 2021). Until now, two potential pathways for the spread of bacteria from the mouth to the colon have been identified. The first route involves continuous swallowing of oral bacteria into the colorectum (oral cavity-alimentary tract-colorectal polyps) (Nakajima et al., 2015; Ou et al., 2022), and the second involves spreading of oral bacteria via bloodstream and systemic circulation (bacteremia) to the colon (oral cavity-circulatory system-colorectal polyps) (Parahitiyawa et al., 2009; Abed et al., 2020). Many studies have demonstrated that oral pathogens migrate to intestinal tissue and may resemble a microbial biofilm. This migration can promote tumorigenesis by changing the tumor microenvironment and inhibiting immune responses, which results in the development of cancer in normally healthy tissue (Tuominen and Rautava, 2021; Yu et al., 2022). However, differences in oral microbial community between patients with polyps and healthy individuals and the pathogenesis of colorectal polyps remain elusive.

Bacteria, which are taxonomically categorized by their phylum, class, order, family, genus, and species, make up most of the gut microbiota. Studies have determined the bacterial microbiome of the human gut through random sequencing of all genes, which mainly included of which *Firmicutes* and *Bacteroides* account for approximately 90% of the intestinal microbiome (Vemuri et al., 2018; Adak and Khan, 2019). *Firmicutes* and *Bacteroidetes* are obligate anaerobic bacteria in the intestinal tract that play important roles in maintaining human health. Disruption of this balance causes inflammatory bowel disease, adenoma, and CRC (Ahlawat and Asha, 2021; Quaglio et al., 2022). *Proteobacteria* is the largest phylum of bacteria, among which γ-Proteobacteria contains many important opportunistic pathogens that can cause changes in the intestinal microenvironment and lead to an overall structural imbalance of the intestinal colonizing flora (Wu et al., 2022; Xu et al., 2022). We discovered that the CP and HC groups had significantly different fecal microbiota abundances. The phyla *Proteobacteria* and *Fusobacteria*, the genera *Fusobacterium* and *Lachnoclostridium*, and the species *R. gnavus*, *P. distasonis*, and *B. ovatus* showed increased abundance in the fecal samples of the CP groups compared to controls, while the genera *Faecalibacterium* and *Dialister* and the species *F. prausnitzii* showed decreased abundance. These results suggested that the fecal microbiota structure in patients with polyps underwent significant changes, and that there was a structural imbalance of intestinal colonization flora with less number of beneficial bacteria and more number of harmful bacteria, consistent with previous research reports (Lo et al., 2022; Zhong et al., 2022; Zhou et al., 2022; Senthakumaran et al., 2023). Therefore, we speculate that the imbalance of intestinal colonization flora may be one of the factors that induce colorectal carcinogenesis.

In this study, we observed that the microorganisms in the fecal samples of the CP group were significantly different from those in controls, and the Shannon and Simpson indices were decreased, consistent with the findings of Ahn et al. (2013) and Ai et al. (2019). Some scholars have theorized that the intestinal flora changes according to the changes in human diet. The loss of less abundant but critical flora leads to destruction of intestinal homeostasis and a decrease in microbial diversity (Sonnenburg and Sonnenburg, 2019). Additionally, the reduced immune response in the diseased tissue may be the cause of the lower bacterial diversity found in the polyps. However, a previous study has reported higher microbial diversity at tumor-associated sites than that in normal tissues (Senthakumaran et al., 2023). This may be the result of intensive flushing of tumors and polyps, which may enrich tissue nutrition and support higher microbial diversity. However, some studies have found no significant difference in α-diversity between healthy mucosa and polyp tissues (Goedert et al., 2015; Chen et al., 2021; Zhong et al., 2022). One study showed higher microbial diversity in the feces of the CP group than in normal mucosa (Li et al., 2022). Most studies used 16S rRNA gene sequencing for microbiome analysis; the difference in results could be due to the target variable region selections, sequencing platform and databases applied, or inconsistent sequencing depth (Zhang et al., 2021).

We constructed a co-occurrence network of differential species in the CP and HC groups. We observed an increase abundance of *L. wadei*, *P. gingivalis*, *F. nucleatum*, and *P. intermedia* in the oral cavity of CP, and identified that *F. nucleatum* was negatively correlated with both *F. prausnitzii* and *P. distasonis*, and *L. wadei* was positively correlated with *P. gingivalis* and *F. nucleatum*, which has not been reported in previous studies. Our results suggest that an increased oral pathogen density contributes to dysbiosis-associated colorectal carcinogenesis. In patients with polyps, the population of butyrate-producing beneficial species that help to maintain intestinal microbiota homeostasis was decreased, and various opportunistic pathogens that can induce inflammatory or metabolic disorders were increased. Gram-negative anaerobic bacteria *P. gingivalis and F. nucleatum* have a number of virulence factors that make them potential pathogens linked to periodontal disease (de Andrade et al., 2019). According to recent studies, *P. gingivalis* was enriched in tissue and fecal samples and was positively correlated with a poor prognosis in CRC patients (Wang et al., 2021; Liu et al., 2022; Ou et al., 2022). They have the ability to accelerate the development of colorectal tumors (Okumura et al., 2021), stimulate the recruitment of tumor-infiltrating myeloid cells, and promote the development of colorectal tumorigenesis through the activation of the PI3K/AKT23 and JAK/STAT324 signaling pathways, as well as inhibition of apoptosis through the inhibition of mitochondrial membrane permeability and cytochrome-c release (Yao et al., 2010; Nakayama et al., 2015; Wang et al., 2021). According to previous studies, *F. nucleatum* promotes the progression of CRC through localization, proliferation, immune suppression, metastasis, and chemoresistance (Abed et al., 2016; Casasanta et al., 2020; Xu et al., 2021). A genus of gram-negative anaerobic bacteria called *Parabacteroides* that frequently colonizes the gastrointestinal tract of many species, *P. distasonis*, has been linked to both pathogenic and beneficial effects on human health (Ezeji et al., 2021). A previous study showed that *P. distasonis* abundance in feces was inversely correlated with the presence of intestinal tumors (Gu et al., 2020). *P. distasonis* could suppress the production of proinflammatory cytokines in a colon cancer cell line and exhibited anti-inflammatory and antitumor properties through downregulation of TLR4/MYD88/Akt signaling and stimulation of apoptosis (Koh et al., 2020). *F. prausnitzii* belonging to the genus *Faecalibacterium* is the major species of the human gut and a significant gut butyrate producer, which has anti-inflammatory properties; reduced abundance of the bacteria has been reported in various intestinal disorders (Lopez-Siles et al., 2017; Leylabadlo et al., 2020). Butyrate is a substance found in the intestine that can reduce gut inflammation and inhibit the growth of colon cancer cells, thus protecting against colon cancer (Zhou et al., 2018). In conclusion, biomarkers identified in this study imply that the majority of the pathogenic and beneficial biomarkers have a significant and widespread influence on the individuals. Recent studies on CRC have identified many fecal microbial markers, and attempts to combine results of markers from oral pathogens, gut microbiome-associated metabolites, FOBT, and FIT have shown good diagnostic performance (Coker et al., 2022; Gao et al., 2022; Zhou et al., 2022; Zwezerijnen-Jiwa et al., 2023). The recent systematic review showed that an AUC of 0.28 to 0.98 for precursor lesions like advanced adenomas and 0.54 to 0.89 for early CRC were observed for the diagnostic performance of fecal bacteria-derived biomarkers. In addition, based on the co-metabolome, diagnostic performance revealed an AUC of 0.69–0.84 for precursor lesions and 0.65–0.93 for early CRC. When combined with clinically validated early detection markers like guaiac FOBT, all models demonstrated improved outcomes. Our study identified salivary and fecal colorectal polyps-enriched bacterial species showing an area under the ROC curve of 0.80 and 0.76, respectively, for distinguishing patients with colorectal polyps from controls, which was increased to 0.82 when combined with salivary and fecal microorganisms, consistent with the above findings. Novel bacterial markers for noninvasive diagnosis of colorectal polyps include *S. thermophilus*, *S. gordonii*, *S. anginosus*, *P. denticola*, *H. parainfluenzae*, *T. forsythia*, *E. nodatum*, *S. sputigena*, and *P. micra*. Salivary and fecal microbiomes have the potential to complement existing screening tools for colorectal polyps. However, on accurate biomarkers for daily clinical practice use, a clear consensus has not yet been reached. The main reasons for this are large variations among studies, including sex, age, diet, antibiotic exposure, location, and samples from different body parts (Lee et al., 2018; Makki et al., 2018; Zwezerijnen-Jiwa et al., 2023). The cost of the full-length 16S rRNA gene sequencing is significantly lower than the colonoscopy and will decrease as technology continues to develop, which takes 2 weeks. In addition, compared with colonoscopy, the method of predicting colorectal polyps by using microbiota is non-invasive. The collection of salivary and fecal samples is relatively easy, noninvasive, and has high patient compliance. Therefore, this method is expected to be further promoted and applied in clinical practice, which can significantly reduce the pressure of medical economy and has a broad market prospect. However, compared with colonoscopy, the accuracy and sensitivity of saliva and fecal microbiota in predicting colorectal polyps need to be further improved. Further studies are needed to verify the results of our study, and other auxiliary diagnostic methods should be further explored.

*S. thermophilus* is one of the most valuable homo-fermentative lactic acid bacteria, which, for a long time, has been widely used as a starter for the production of fermented dairy products (Cui et al., 2017). The important production characteristics of *S. thermophilus* were proteolytic enzymes, extracellular polysaccharide as well as acidifying capacity etc., have an important effect on the quality of dairy products (Cui et al., 2016). *S. thermophilus* has been shown to protect the gastrointestinal epithelium from entero-invasive *Escherichia coli*, improve somatic growth in infants, and reduce the severity and duration of acute diarrhea in young infants (Thibault et al., 2004; Corrêa et al., 2005). Studies have demonstrated that *S. thermophilus* is depleted in CRC patients’ gut compared with healthy controls (Dai et al., 2018). The cluster analysis in our study found that *S. thermophilus* was widely present in salivary samples and less abundant in fecal samples. Association analysis of salivary and fecal samples from the CP group found a weak positive association between *S. thermophilus* and harmful bacterium of colorectal polyps (*C. concisus*, *F. nucleatum*, *P. intermedia* and so on). The results of our study were not consistent with previous studies (Thibault et al., 2004; Corrêa et al., 2005; Dai et al., 2018), which may be caused by the fact that we included salivary and fecal microorganism at the same time in the association network, and did not use relatively correct ways that allow to distinguish *S. thermophilus* strains belonging to salivary samples or fecal samples. When attempting to recover *S. thermophilus* from fecal samples, one often neglected but critical aspect is its phylogenetic similarity with two other closely related species, namely *Streptococcus salivarius* and *Streptococcus vestibularis* (Delorme et al., 2015). These three species, which are genetically very similar (Delorme et al., 2015), belong to the *S. salivarius* subgroup of viridians streptococci. *S. salivarius* and *S. vestibularis* are commensal bacteria of the oral and gastrointestinal cavities and of the genital tract (Kawamura et al., 1995). In addition, the prevalence and abundance of *S. thermophilus* in fecal samples is generally low and linked to age, lifestyle, and geography (Martinović et al., 2020). Therefore, the relationship between *S. thermophilus* and colorectal polyps in fecal samples needs to be further verified.

This study has several strengths. First, the family members of the CP group were considered as controls. All studied groups were characterized by a similar distribution of age, sex, BMI, education level, smoking history, frequency of tooth brushing per day, and frequency of oral visits. Therefore, the groups were comparable as they shared similar history. Another strength lies in the collection of samples. We collected salivary samples, consistent with previous studies that have confirmed saliva as an ideal source for sampling oral microorganism associated with cancer risk, and a superior sampling site to acquire microbial DNA sequencing in the research of oral microbiota (Slots and Slots, 2011; Belstrøm et al., 2016; Hall et al., 2017; Fan et al., 2018). Moreover, we used a special fecal collection box and fecal preservation solution to reduce its contamination and stabilize nucleic acids present in stools. Furthermore, all salivary and fecal samples were shipped on ice and frozen at −80°C within 2–4 h. Previous research has shown that the microbial community in fecal samples stored at room temperature for up to 24 h is largely unaffected (Sinha et al., 2016), and that up to 72 h of storage at 4°C results in no appreciable changes in microbial diversity or composition. Next, screening of patients was performed by complete colonoscopy, which is regarded as the gold standard examination for the presence or absence of colorectal polyps (Choo et al., 2015). Additionally, the overall microbial composition may not be significantly changed by keeping fecal samples at −80°C for an extended period of time (Carroll et al., 2012; Hale et al., 2015).

The limitations of this study were included below. First of all, because the case–control study only provides a “snapshot” of the examined diseases without establishing the temporal relationship between the exposure and the outcome, our findings are unable to explain any causal relationship between salivary and fecal microorganisms. Second, only one hospital served as the source for recruiting the participants, the results may not be representative of the general population, and selection bias may exist. Thirdly, pathological type, location, size, and number of polyps were not analyzed further in this study. Therefore, prospective, and controlled longitudinal studies on patients with colorectal polyps are necessary to illuminate the role of microbiota in colorectal polyps and their mechanism of carcinogenesis and progression to CRC to provide more powerful evidence for the diagnosis and treatment of CRC.

## 5. Conclusion

In conclusion, we studied the composition and structure of the salivary and fecal microbiota in patients with colorectal polyps using full-length 16S rRNA gene sequencing. The results showed significant differences in the salivary and fecal microbiota between patients with colorectal polyps and healthy controls. There was a structural imbalance of the colonizing flora between beneficial bacteria, including *P. nanceiensis* and *F. prausnitzii*, and harmful bacteria (increased abundance), including *L. wadei*, *P. gingivalis*, *F. nucleatum*, *P. intermedia*, *R. gnavus*, *P. distasonis*, and *B. ovatus*. Our study showed that salivary and fecal microbiome-derived biomarkers could be used to improve the current screening methods for colorectal polyps. These discoveries may help with early screening, detection, and diagnosis of colorectal polyps, reveal information about disease-specific and cross-disease microbial patterns, and have significant clinical implications by elevating patient quality of life and lessening financial strain on the healthcare system. However, more research is needed to fully clarify the genetic and biological mechanisms underlying the link between the microbiota and colorectal polyps.

## Data availability statement

The datasets presented in this study can be found in online repositories. The names of the repository/repositories and accession number(s) can be found below: BioProject, PRJNA957055.

## Ethics statement

The studies involving human participants were reviewed and approved by the Ethics Committee of the Shanghai Fifth People’s Hospital, Fudan University. The patients/participants provided their written informed consent to participate in this study.

## Author contributions

LZ: conceptualization, data curation, formal analysis, investigation, methodology, project administration, resources, and writing-original draft. ZF: investigation, methodology, data curation, formal analysis, project administration, resources, and writing-original draft. YL: methodology, project administration, resources, and software. CuL: methodology, project administration, resources, and software. ChL: investigation, methodology, data curation, formal analysis, project administration, and resources. YH: investigation, methodology, data curation, formal analysis, project administration, and resources. MF: conceptualization, data curation, formal analysis, methodology, project administration, resources, supervision, and writing-review and editing. LS: conceptualization, funding acquisition, methodology, project administration, supervision, and writing-review and editing. All authors contributed to the article and approved the submitted version.

## Funding

This work was supported by the Natural Science Research Funds of Minhang District, Shanghai (No. 2021MHZ083); high-level professional physician training program of Minhang District (No. 2020MZYS08), Medical education research project of Chinese Medical Association Medical Education Branch and Chinese Higher Education Association Medical Education Professional Committee (No. 2020B-N02163).

## Conflict of interest

The authors declare that the research was conducted in the absence of any commercial or financial relationships that could be construed as a potential conflict of interest.

## Publisher’s note

All claims expressed in this article are solely those of the authors and do not necessarily represent those of their affiliated organizations, or those of the publisher, the editors and the reviewers. Any product that may be evaluated in this article, or claim that may be made by its manufacturer, is not guaranteed or endorsed by the publisher.

## Footnotes

^1^https://docs.qiime2.org/2019.4/tutorials/
