# Supplementary material for: Salivary and fecal microbiota: potential new biomarkers for early screening of colorectal polyps
Source: Front Microbiol. 2023 Aug 16;14:1182346. doi: 10.3389/fmicb.2023.1182346 (PMC10467446; doi:10.3389/fmicb.2023.1182346)
Supplement: Supplementary file 1 [file Table_1.docx]

**Supplementary Tables**

**Supplementary Table 1 The importance of salivary microbiota in predicting for colorectal polyps.**

| Species | Importance |
| --- | --- |
| Parvimonas_micra | 0.06697449 |
| Streptococcus_gordonii | 0.051089119 |
| Streptococcus_anginosus | 0.050966428 |
| Prevotella_denticola | 0.050875732 |
| Tannerella_forsythia | 0.049110098 |
| Prevotella_nanceiensis | 0.047985819 |
| Prevotella_intermedia | 0.044639843 |
| Leptotrichia_wadei | 0.042663915 |
| Leptotrichia_sp._oral_taxon_498 | 0.038986722 |
| Fusobacterium_nucleatum | 0.036979851 |
| Megasphaera_micronuciformis | 0.035907186 |
| Treponema_socranskii | 0.033749309 |
| Filifactor_alocis | 0.031802357 |
| Campylobacter_gracilis | 0.031694799 |
| Dialister_pneumosintes | 0.030478715 |
| Selenomonas_sputigena | 0.029935254 |
| _Eubacterium__nodatum | 0.028777317 |
| Selenomonas_noxia | 0.027538468 |
| Neisseria_flavescens | 0.026498568 |
| Prevotella_veroralis | 0.025077975 |
| Treponema_denticola | 0.024571911 |
| Porphyromonas_gingivalis | 0.023447171 |
| Prevotella_oralis | 0.022706052 |
| Leptotrichia_sp._oral_taxon_847 | 0.021973901 |
| Dialister_invisus | 0.021756973 |
| Aggregatibacter_segnis | 0.021368992 |
| Streptococcus_cristatus | 0.018964235 |
| Eggerthia_catenaformis | 0.018031413 |
| Prevotella_baroniae | 0.017690008 |
| Prevotella_buccae | 0.013155565 |
| Prevotella_dentalis | 0.009045659 |
| Bacteroides_heparinolyticus | 0.005556157 |

**Supplementary Table 2 The importance of salivary and fecal microbiota in predicting for colorectal polyps.**

| Species | Importance |
| --- | --- |
| Faecalibacterium_sp. | 0.152647108 |
| Faecalibacterium_prausnitzii | 0.12568748 |
| Parabacteroides_distasonis | 0.114220877 |
| Ruminococcus__gnavus | 0.096547258 |
| Bacteroides_ovatus | 0.095532379 |
| Clostridium__symbiosum | 0.084833764 |
| Adlercreutzia_equolifaciens | 0.073774502 |
| Erysipelatoclostridium_ramosum | 0.0715736 |
| Ruminococcus_bicirculans | 0.062252676 |
| Oxalobacter_formigenes | 0.06158323 |
| Citrobacter_freundii | 0.061347126 |

**Supplementary Table 3 The importance of salivary and fecal microbiota in predicting for colorectal polyps.**

| Species | Importance |
| --- | --- |
| Streptococcus_thermophilus | 0.062329905 |
| Streptococcus_gordonii | 0.050936598 |
| Streptococcus_anginosus | 0.045965181 |
| Prevotella_denticola | 0.045817179 |
| Haemophilus_parainfluenzae | 0.043259805 |
| Tannerella_forsythia | 0.042755965 |
| Eubacterium__nodatum | 0.042509692 |
| Selenomonas_sputigena | 0.041892382 |
| Parvimonas_micra | 0.040444221 |
| Faecalibacterium_sp. | 0.039821134 |
| Granulicatella_adiacens | 0.034444155 |
| Parabacteroides_distasonis | 0.03402558 |
| _Ruminococcus__gnavus | 0.032560747 |
| Bacteroides_dorei | 0.031374147 |
| Phascolarctobacterium_faecium | 0.031225168 |
| Solobacterium_moorei | 0.029882626 |
| Streptococcus_pneumoniae | 0.029828573 |
| Bacteroides_ovatus | 0.029295285 |
| Faecalibacterium_prausnitzii | 0.029053218 |
| Dialister_invisus | 0.029025937 |
| Flavonifractor_plautii | 0.028674698 |
| Escherichia_coli | 0.028258712 |
| Collinsella_aerofaciens | 0.026027326 |
| Bacteroides_vulgatus | 0.025264535 |
| Erysipelatoclostridium_ramosum | 0.024961627 |
| Blautia_wexlerae | 0.022582319 |
| Bacteroides_uniformis | 0.022504877 |
| _Clostridium__symbiosum | 0.021256993 |
| Enterobacter_hormaechei | 0.020054616 |
| Ruminococcus_bicirculans | 0.013966801 |

**Supplementary Table 4 Association network of salivary and fecal microbial species in patients with colorectal polyps**

|  |  | *Faecalibacterium prausnitzii* | *Ruminococcus gnavus* | *Parabacteroides distasonis* | *Leptotrichia wadei* | *Prevotella nanceiensis* | *Bacteroides ovatus* | *Porphyromonas gingivalis* | *Fusobacterium nucleatum* | *Prevotella intermedia* |
| --- | --- | --- | --- | --- | --- | --- | --- | --- | --- | --- |
| *Faecalibacterium prausnitzii* | R | 1 | −0.101 | 0.062 | −0.217 | −0.108 | 0.271* | −0.181 | −0.265* | −0.209 |
|  | P | - | 0.421 | 0.621 | 0.080 | 0.390 | 0.028 | 0.147 | 0.032 | 0.091 |
| *Ruminococcus gnavus* | R | −0.101 | 1 | 0.504** | −0.150 | −0.074 | 0.094 | −0.125 | −0.183 | −0.144 |
|  | P | 0.421 | - | 0.000 | 0.231 | 0.554 | 0.455 | 0.319 | 0.141 | 0.247 |
| *Parabacteroides distasonis* | R | 0.062 | 0.504** | 1 | −0.241 | −0.120 | 0.553** | −0.201 | -0.296* | −0.233 |
|  | P | 0.621 | 0.000 | - | 0.051 | 0.338 | 0.000 | 0.105 | 0.016 | 0.060 |
| *Leptotrichia wadei* | R | −0.217 | −0.150 | −0.241 | 1 | −0.045 | −0.180 | 0.437** | 0.342** | 0.087 |
|  | P | 0.080 | 0.231 | 0.051 | - | 0.718 | 0.148 | 0.000 | 0.005 | 0.489 |
| *Prevotella nanceiensis* | R | −0.108 | −0.074 | −0.120 | −0.045 | 1 | −0.089 | 0.026 | 0.166 | 0.292* |
|  | P | 0.390 | 0.554 | 0.338 | 0.718 | - | 0.476 | 0.834 | 0.182 | 0.017 |
| *Bacteroides ovatus* | R | 0.271* | 0.094 | 0.553** | −0.180 | −0.089 | 1 | −0.150 | −0.221 | −0.174 |
|  | P | 0.028 | 0.455 | 0.000 | 0.148 | 0.476 | - | 0.229 | 0.075 | 0.163 |
| *Porphyromonas gingivalis* | R | −0.181 | −0.125 | −0.201 | 0.437** | 0.026 | −0.150 | 1 | 0.522** | 0.354** |
|  | P | 0.147 | 0.319 | 0.105 | 0.000 | 0.834 | 0.229 | - | 0.000 | 0.004 |
| *Fusobacterium nucleatum* | R | −0.265* | −0.183 | −0.296* | 0.342** | 0.166 | −0.221 | 0.522** | 1 | 0.525** |
|  | P | 0.032 | 0.141 | 0.016 | 0.005 | 0.182 | 0.075 | 0.000 | - | 0.000 |
| *Prevotella intermedia* | R | −0.209 | −0.144 | −0.233 | 0.087 | 0.292* | −0.174 | 0.354** | 0.525** | 1 |
|  | P | 0.091 | 0.247 | 0.060 | 0.489 | 0.017 | 0.163 | 0.004 | 0.000 | - |

** *P*-value <0.01

* *P*-value <0.05
